# Differential transport function of lymphatic vessels in the rat tail model and the long-term effects of Indocyanine Green as assessed with near-infrared imaging

**DOI:** 10.3389/fphys.2013.00215

**Published:** 2013-08-15

**Authors:** Michael Weiler, J. Brandon Dixon

**Affiliations:** Wallace H. Coulter Department of Biomedical Engineering, George W. Woodruff School of Mechanical Engineering, Parker H. Petit Institute for Bioengineering and Bioscience, Georgia Institute of TechnologyAtlanta, GA, USA

**Keywords:** indocyanine green, ICG, lymphatic, near-infrared imaging, lymphatic imaging, lymphatic function

## Abstract

**Introduction:** Near-infrared (NIR) imaging has emerged as a novel imaging modality for assessing lymphatic function *in vivo*. While the technique has provided quantitative data previously unavailable, questions remain in regards to the spatiotemporal capabilities of the approach. We address three of the more important issues here using the rodent tail, one of the most widely utilized *in vivo* model systems in the lymphatic literature. Specifically we demonstrate (1) the transient vs. steady state response of lymphatics to tracer injection, (2) the functional characteristics of multiple collecting vessels draining the same tissue space in parallel, and (3) the long-term consequences of fluorescent tracers on lymphatic function to repeated functional measurements.

**Methods**: Rat tails were imaged with NIR and metrics of function were calculated for both collecting vessels that drain the tail. A nitric oxide donor cream (GTNO) was applied to the tail. Additionally, two different NIR dyes, indocyanine green (ICG) and LI-COR IRDye 800CW PEG, were utilized for function imaging at the time of initial injection and at 1, 2, and 4 week follow-up time points after which both draining lymph nodes were harvested.

**Results and Discussion**: Significant differences were found between the two collecting vessels such that the vessel first showing fluorescence (dominant) produced enhanced functional metrics compared to the second vessel (non-dominant). GTNO significantly reduced lymphatic function in the non-dominant vessel compared to the dominant. ICG remained visible in the tail for 2 weeks after injection and was accompanied by significant losses in lymphatic function and enlarged draining lymph nodes. The Licor tracer also remained visible for 2 weeks. However, the dye produced significantly lower effects on lymphatic function than ICG, and lymph nodes were not enlarged at any time point, suggesting that this may be a more appropriate contrast agent for longitudinal lymphatic imaging.

## Introduction

The lymphatic vasculature is present in nearly every tissue of the body to serve essential functions in fluid homeostasis (Dongaonkar et al., 2009; Nipper and Dixon, 2011), immune cell trafficking (Swartz et al., 2008; Weber et al., 2013), and lipid transport (Dixon, 2010), and it has been implicated in the progression of several diseases (Rockson, 2008; Tammela and Alitalo, 2010). Despite the critical roles that this system performs, very little is known about the lymphatic vasculature in comparison to the blood vasculature, which can be attributed, in part, to the historic difficulty associated with imaging lymphatic vessels (Sharma et al., 2008). With the growing interest in studying lymphatics, near-infrared (NIR) imaging has emerged as a novel lymphatic imaging modality to simultaneously improve spatial resolution to visualize small initial lymphatics and increase temporal resolution to capture the dynamic lymphatic pump function responsible for fluid propulsion.

To date, NIR lymphatic imaging has produced exciting results quantifying lymphatic transport (Rasmussen et al., 2009), illustrating differences in lymphatic architecture between normal and severe disease cases (Unno et al., 2007), showing differences in function and architecture due to genetic mutation (Burrows et al., 2013), and assessing changes in lymphatic function in response to various manipulations (Weiler et al., 2012; Blum et al., 2013). However, the technique remains immature, and the field is still learning to understand and interpret the wealth of data NIR functional lymphatic imaging can uniquely provide compared to other modalities. In particular, recent work has suggested functional differences between two collecting vessels in a rodent hind limb (Proulx et al., 2013), but no studies have specifically examined the differential transport abilities of multiple collecting vessels draining a single tissue space. Such an analysis is a necessary advancement of NIR functional lymphatic imaging, which has historically focused on quantification of only a single vessel, to better understand the physiology of draining lymphatic networks at the tissue level.

Therefore, we have chosen to simultaneously characterize the two collecting vessels in the rat tail model using NIR lymphatic imaging. The rat tail provides the simplest model of lymphatic network drainage for NIR imaging purposes due to the simple geometry and the consistent position of the two collecting vessels. Using the rodent tail also allows comparisons to many previous studies as it has been one of the most widely used models in lymphatic research, providing insight into basic lymphatic physiology and lymph flow (Leu et al., 1994; Weiler et al., 2012), lymphangiogenesis (Boardman and Swartz, 2003; Yoon et al., 2003; Goldman et al., 2007; Clavin et al., 2008), and lymphedema pathology (Rutkowski et al., 2006; Tabibiazar et al., 2006; Zampell et al., 2012). The results of this characterization will establish a framework by which future lymphatic research can be performed using NIR imaging in the tail model and will enhance our understanding of differential vessel function.

Additional controversy also remains regarding the most appropriate fluorophore for NIR lymphatic imaging. Some studies are beginning to explore novel probes with higher quantum yields that specifically target lymphatic vessels (Proulx et al., 2010, 2013; Davies-Venn et al., 2011), but the most commonly used probe to date has been indocyanine green (ICG). Despite the low quantum yield of the molecule, ICG remains the hallmark of NIR lymphatic imaging because it is FDA approved for use in humans and represents the most likely probe for the development of a point-of-care diagnostic. However, the literature is mixed regarding the effects of ICG on lymphatic function.

It has been shown in isolated lymphatic vessels that ICG inhibits vessel contraction in a dose-dependent manner and continues to alter function even beyond complete washout from the vessel (Gashev et al., 2010). Given that near-infrared fluorophores have been shown to accumulate in the intracellular space (Frangioni, 2003), and ICG, in particular, has been shown to exhibit extremely cumulative cellular uptake (Fickweiler et al., 1997; Abels et al., 2000), we hypothesize that ICG is retained in the tissue space and contributes to a decrease in lymphatic function for an extended period of time following initial injection. A follow-up study using NIR imaging was unable to detect changes in lymphatic function after ICG injections of various concentrations (Aldrich et al., 2012), but the study only examined this phenomenon at one time point. Therefore, in this study we will first examine the retention of ICG in the tissue space of the rat tail and measure lymphatic function with NIR imaging beyond the duration of retention to analyze the time course changes in lymphatic function following initial ICG injection. For comparison purposes, we will also perform this analysis with a competing NIR fluorophore, the LI-COR IRDye 800CW PEG. The results of this study will contribute to a further characterization of the rat tail model for NIR lymphatic imaging and will inform future studies involving multiple, repeat injections of NIR probes in this model.

## Methods

### Hardware configuration

**Figure 1:**
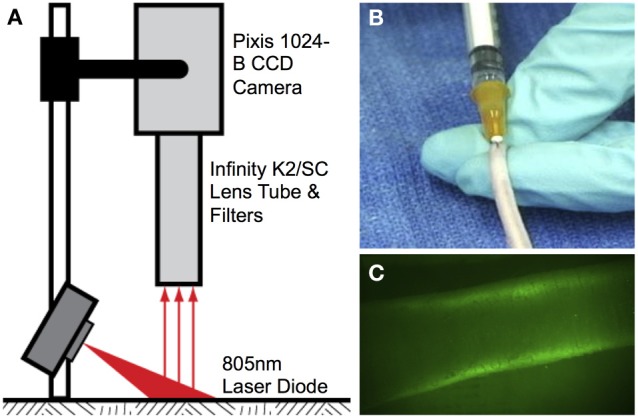
**Near-infrared lymphatic imaging setup. (A)** Schematic of near-infrared imaging hardware. **(B)** Intradermal tail injection of near-infrared fluorophore. **(C)** Example of imaging window 10 cm proximal to injection site showing fluorescence uptake in the two collecting lymphatic vessels.

NIR imaging hardware (Figure 1A) consisted of a 150 mW 808 nm laser diode (Thorlabs part no. M9-808-0150) powered by accompanying diode driver and temperature control boxes to provide excitation light. A 20° beam diffuser (Thorlabs part no. ED1-C20) was mounted in front of the diode to achieve a uniform excitation field. Fluorescence emission centered at 840 nm was captured using a PIXIS 1024B back-illuminated CCD camera (Princeton Instruments) with an attached Infinity K2/SC video microscope lens (Edmund Optics) and a bandpass filter (CW:840 nm, FWHM:15 nm, Omega Optical). NIR images were recorded via a custom LabVIEW (National Instruments) image acquisition code.

### NIR imaging procedure

Lymphatic function was quantified *in vivo* in the tail of 6-week-old female Sprague Dawley rats (Charles River Laboratories, Wilmington, MA) according to procedures approved by the Georgia Institute of Technology IACUC Review Board and performed in our laboratory previously (Weiler et al., 2012). All animals were first anesthetized using an intramuscular injection of Fentanyl (0.12 mg/kg), Droperidol (6 mg/kg), and Diazepam (2.5 mg/kg given 10 min after Fentanyl/Droperidol). A 10 μL fluorophore solution of either ICG (Across Organics) pre-mixed with bovine serum albumin (BSA) (MP Biomedicals, New Zealand) at a concentration of 150 μg/mL ICG and 60 mg/mL BSA or LI-COR IRDye 800CW PEG was then injected intradermally into the tip of the tail for fluorescence imaging (Figure 1B). An injection volume of 10 μL was chosen based upon past success in rodent models, so as to not overload the lymphatics with unnecessary fluid volume while at the same time providing enough tracer for sufficient detection (Kwon and Sevick-Muraca, 2010; Weiler et al., 2012). The injection was given at an entry angle of approximately 10° to an approximate depth of 1 mm to specifically target the lymphatic vasculature. Care was taken to position the injection as close to the midline of the tail as possible to avoid favoring one collecting vessel over the other. The excitation source and the field of view of the CCD emission detector were centered on the rats' tail 10 cm downstream (toward the base of the tail) from the injection site at the tip of tail (Figure 1C). This location ensured that only the downstream collecting lymphatics would be visualized so as to avoid any potential complications from fluorescence uptake by initial lymphatics. The small volume of fluid injection and the use of NIR to enhance tissue penetration ensures that only fluorescence in the deeper collecting lymphatics is visible downstream of the injection site. The animals were imaged continuously from the time of injection until 20 min post-injection with a 50 ms exposure time.

### Quantifying lymphatic function

**Figure 2:**
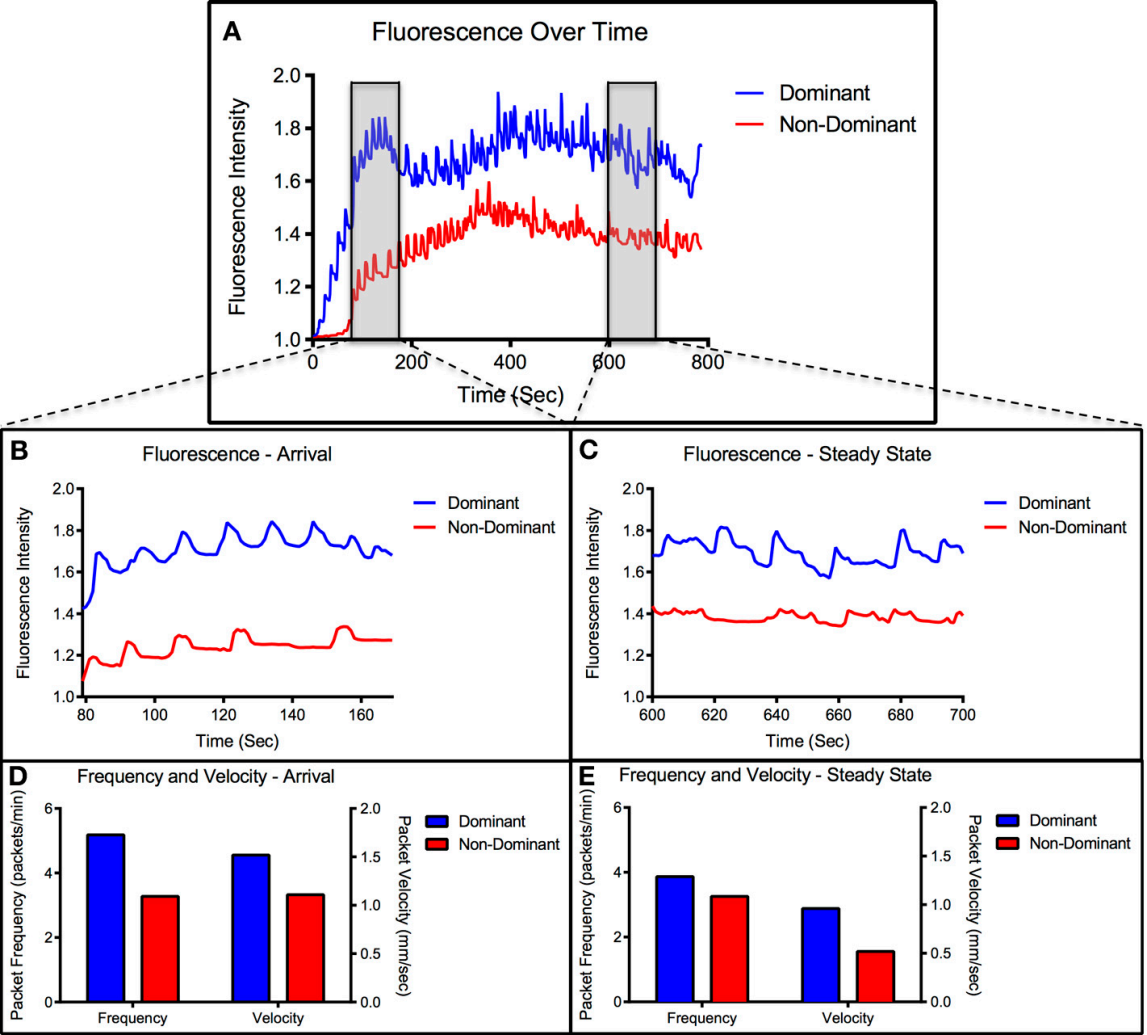
**Representative vessel transport characteristics. (A)** Representative data set showing fluorescence over time for dominant and non-dominant vessel. Arrival and steady-state segments are highlighted in the gray boxes. **(B)** Close-up view of intensity signal during arrival segment. **(C)** Close-up view of intensity signal during steady-state segment. **(D)** Packet frequency and packet velocity during arrival segment. **(E)** Packet frequency and packet velocity during steady-state segment.

To evaluate lymphatic function, three parameters were measured: the time necessary for the bolus injection of ICG to travel the 10 cm distance from injection site to emission recording site (transport time), the average velocity of the packets traveling through the field of view of the recording site, and the average frequency of packets passing through the field of view according to previously published methods (Weiler et al., 2012). In order to characterize the individual function of the two collecting lymphatic vessels of the rat tail and to quantify the relationship between them, the three lymphatic function metrics were separately calculated for the two vessels of the tail. For the purposes of this analysis, the vessel in which fluorescence first arrived (marked by a 20% increase in intensity) was defined as the dominant vessel and the other vessel was defined as non-dominant. An example of intensity plots in the two vessels can be seen in Figure 2A. To evaluate differences in the transient response to fluid injection from the steady state response, lymphatic function metrics were calculated in two separate 100-s segments after injection: the arrival segment beginning 60 s after fluorescence arrival in each vessel and the steady state segment beginning 10 min after injection. A representative example of the functional metrics calculated for both segments in a healthy animal is shown in Figures 2B–E. A paired, two-tail *t*-test with a Bonferonni correction for multiple comparisons was used to test for significance with α = 0.05.

### Lymphatic imaging in response to nitric oxide treatment

A nitric oxide (NO) donor cream (glyceryl trinitrate ointment, GTNO) was applied to the tail as a second experimental condition to observe the effects of NO on the lymphatic function metrics of the two vessels (*n* = 3). GTNO was applied to the whole tail one-minute prior to injection. Lymphatic function was calculated for both collecting vessels in the same manner described above. Comparisons between normal and GTNO-treated animals were checked for significance using an unpaired, two-tail *t*-test with a Bonferroni correction and α = 0.05. Significance within the GTNO treatment group was analyzed using a paired, two-tail *t*-test with a Bonferroni correction and α = 0.05.

### Time-course analysis of ICG

To characterize the long-term effects of ICG on lymphatic function, a time course study was performed. Initially, a single 10 μL intradermal ICG injection (150 μg/mL ICG and 60 mg/mL BSA) was given in the tip of the tail (*n* = 4) and fluorescence measurements were taken every 2 days to track ICG retention.

Based upon the results of the retention study, a second study was performed to monitor changes in lymphatic function during the ICG retention period, which is depicted graphically in Table 1. All animals in this group were given an initial 10 μL intradermal ICG injection (150 μg/mL ICG and 600 mg/mL BSA) in the tip of the tail at week 0 and baseline lymphatic function measurements were recorded for both collecting vessels. The animals were divided into three treatment groups with follow-up 10 μL intradermal ICG injections and functional measurements at 1, 2, and 4-week time points (*n* = 4). Follow-up injections were given as close to the original injection site as possible with particular care to keep the injection at the midline of the tail. Definitions of dominant and non-dominant vessels were made based upon week 0 measurements and remained consistently defined throughout follow-up screenings regardless of follow-up transport times (although the dominant vessel continued to produce the fastest transport times in nearly 90% of cases). Control animals were given a 10 μL intradermal BSA injection in the tip of the tail at week 0 with follow-up 10 μL intradermal ICG injections and functional measurements at the same three time points (*n* = 4). Comparisons of function between time points was checked for significance using a paired, one-tail *t*-test with α = 0.05.

**Table 1:** **ICG time-course experimental setup**.

		**Week 0**	**Week 1**	**Week 2**	**Week 4**
Group 1	Treatment	ICG/IR dye injection	ICG/IR dye injection		
			LN harvest		
	Control	BSA injection	ICG/IR dye injection		
			LN harvest		
Group 2	Treatment	ICG/IR dye injection		ICG/IR dye injection	
				LN harvest	
	Control	BSA injection		ICG/IR dye injection	
				LN harvest	
Group 3	Treatment	ICG/IR dye injection			ICG/IR dye injection
					LN harvest
	Control	BSA injection			ICG/IR dye injection
					LN harvest

Animals were divided into three groups for follow-up imaging at 1, 2, and 4 weeks. All animals were given a 10 μL intradermal injection of either ICG, IR Dye, or BSA at week 0, and function metrics were recorded as a baseline for treatment animals. During the follow-up session for each group, the animals were given a 10 μL fluorophore injection for lymphatic function measurements and lymph nodes were harvested.

A third set of animals was used to observe the effects ICG on lymph node size. All animals were again given a single 10 μL intradermal ICG injection in the tip of the tail. The sciatic lymph node (nearest draining node) was harvested for both collecting vessels without any additional follow-up injections at the week 0, 1, 2, and 4 time points (*n* = 3). Control animals were given a 10 μL intradermal BSA injection in the tip of the tail and lymph nodes were harvested at each time point (*n* = 3) without any follow-up injections. Lymph node size was calculated using projected two-dimensional area from microscopy images. We attempted to take fluorescence images to observe ICG retention in the nodes, but fluorescence was only detectable at the week 0 time point. Comparisons of lymph node size were checked for significance using an unpaired, two-tail *t*-test with a Bonferroni correction and α = 0.05.

In order to compare the results of ICG to another popular NIR fluorophore, the same procedure outlined above was also performed using the LI-COR IRDye 800CW PEG including retention (*n* = 4), time course functional measurements (*n* = 4), and lymph node harvesting (*n* = 3).

## Results

### Spatial and temporal characterization of lymphatic function in the tail

Figure 2 shows a representative example of fluorescence intensity measurements over time and packet frequency and velocity measured during the arrival and steady-state segments. We have denoted the first arrival vessel as dominant and the other as non-dominant. All imaging sessions showed at least a 2 s difference in fluorescence arrival times between the two vessels with greater than 70% of cases showing a difference of 20 s or more. The dominant vessel nearly always produced higher intensity values than the non-dominant vessel throughout the imaging session (Figure 2A) and remained consistent across multiple injections in the majority of animals.

**Figure 3:**
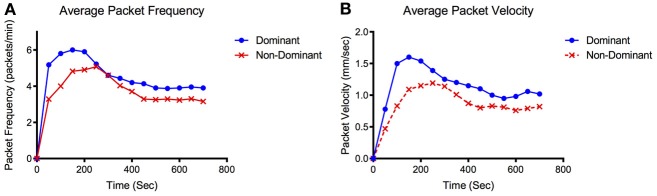
**Representative packet frequency and velocity tracings.** Representative data set showing **(A)** average packet frequency and **(B)** average packet velocity for the dominant and non-dominant vessel over time.

**Figure 4:**
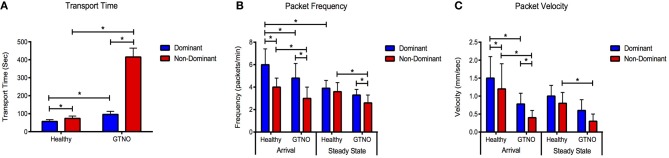
**Lymphatic function metrics in healthy and GTNO-treated cases.** Compiled data for **(A)** transport time, **(B)** packet frequency, and **(C)** packet velocity for the dominant and non-dominant vessel during arrival and steady-state segments in healthy and GTNO-treated animals (*n* = 3). Error bars represent standard deviation. ^*^p < 0.01.

During the arrival period the dominant vessel exhibited a rapid increase in fluorescence while the non-dominant vessel usually produced a more gradual rise in fluorescence. Packet frequency and velocity in both vessels peaked during or shortly after the arrival stage. The values then tapered off toward a lower, constant value during the steady-state period with the dominant vessel constantly producing larger or equal frequencies and velocities than the non-dominant vessel (Figures 2D,E, 3). The dominant vessel produced significantly reduced transport times, and significantly increased packet frequencies and packet velocities compared to the non-dominant vessel in the arrival segment, but there were no significant differences in the three functional metrics between the two vessels in the steady-state pumping period (Figure 4). Packet frequencies and packet velocities were significantly higher during the arrival stage than the steady-state period in the dominant vessel, but not in the non-dominant vessel.

### Differential effects of nitric oxide on lymphatic function

The application of GTNO significantly increased transport times in both vessels compared to healthy animals as we have reported previously (Weiler et al., 2012), but a much larger increase was seen in the non-dominant vessel in which transport times increased nearly 6-fold (Figure 4). NO-mediated vessels also showed significantly decreased packet frequency and packet velocity in both vessels during the arrival period and in the non-dominant vessel during the steady state period.

### Consequences of ICG retention on lymphatic function

**Figure 5:**
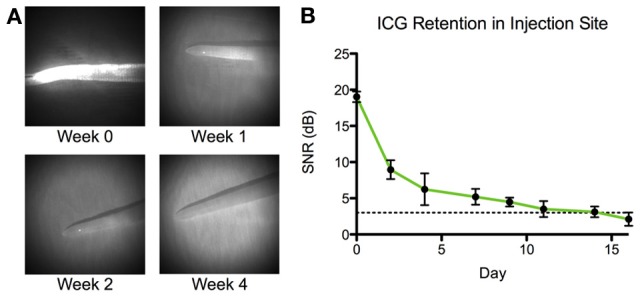
**ICG is retained in tissue space for 2 weeks. (A)** Representative images of the tip of a rat tail at four time points: immediately following ICG injection (week 0) and 1, 2, and 4 weeks after injection. **(B)** Average signal-to-noise ratio (SNR) of ICG in the tip of the rat tail (*n* = 4). Dotted line represents limit of detection at 3 dB. Error bars represent standard deviation.

**Figure 6:**
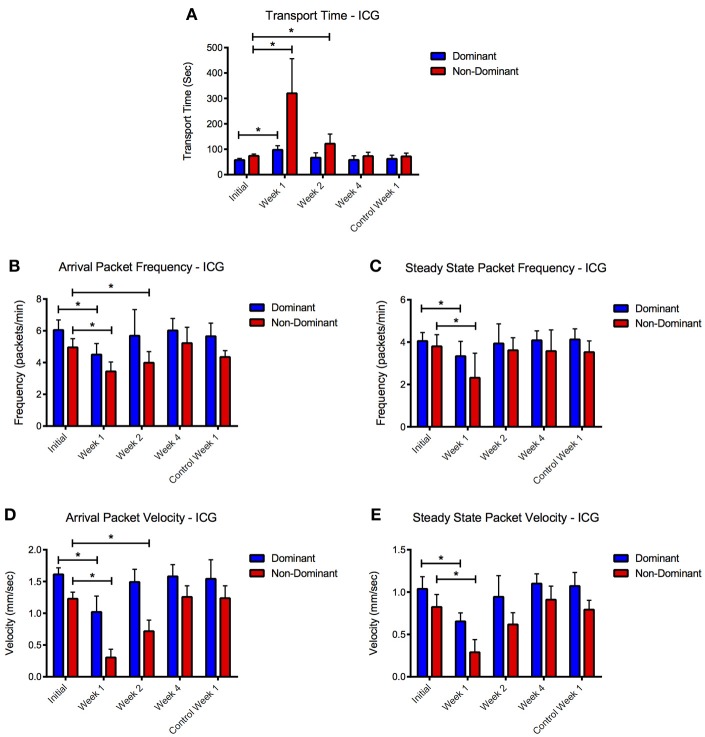
**ICG reduces lymphatic function 1 and 2 weeks after initial injection.** Compiled **(A)** transport time, packet frequency during **(B)** arrival and steady-state **(C)**, and packet velocity during **(D)** arrival and **(E)** steady-state periods for dominant and non-dominant vessels during the four time points and for the control animals at week 1. Error bars represent standard deviation. ^*^p < 0.05.

ICG remained visible (as defined by a signal to noise ratio greater than 3 dB) in the tissue space for more than 2 weeks after initial injection (Figure 5). NIR lymphatic function measurements after follow-up ICG injections of 1 week showed significant reductions in all three lymphatic function metrics in both vessels during the arrival and steady state segments (Figure 6). Transport time remained significantly elevated in the non-dominant vessel at the week 2 time point. Packet frequency and velocity likewise remained elevated in the non-dominant vessel at week 2, but only during the arrival period. No significant differences in function were observed at week 4. Control animals did not produce significant differences in lymphatic function at any time point.

**Figure 7:**
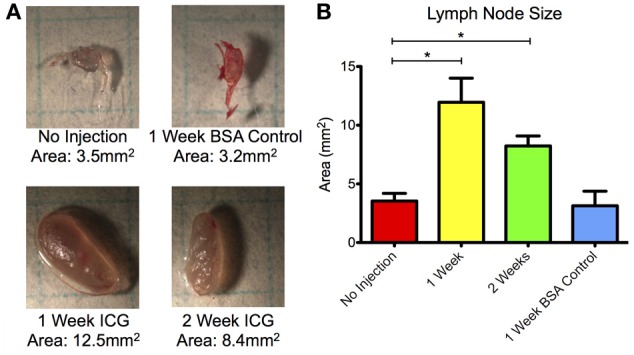
**Draining lymph nodes enlarge 1 and 2 weeks after ICG injection. (A)** Representative microscopy images of sciatic lymph nodes without injection, 1 week after BSA control injection, and 1 and 2 weeks after ICG injection. Grid squares = 25 mm^2^. **(B)** Projected two-dimensional area of lymph nodes prior to injection, 1 and 2 weeks after ICG injection, and 1 week after control BSA injection. Data is compiled for both sciatic lymph nodes at each time point. Error bars represent standard deviation. ^*^p < 0.001.

Lymph nodes were also significantly enlarged in both vessels at the 1 and 2 week time points with more than a 350% increase in projected two-dimensional area at week 1 and almost a 200% increase at week 2 (Figure 7). Lymph nodes harvested at week 4 were not significantly enlarged from their baseline values. Control animals did not produce significant differences in lymph node size at any time point.

**Figure 8:**
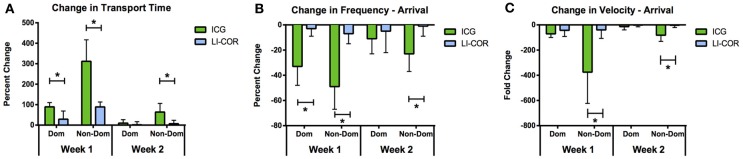
**LI-COR IRDye 800CW PEG reduces lymphatic function less than ICG.** Percent change in **(A)** transport time, **(B)** packet frequency, and **(C)** packet velocity between initial measurement and 1 and 2 week follow-up time points for ICG and LI-COR IRDye 800CW PEG. Error bars represent standard deviation. ^*^p < 0.01.

The LI-COR IRDye 800CW PEG likewise remained visible in the rat collecting lymphatics for approximately 2 weeks. However, the only function metric observed to be significantly different during the follow-up sessions was an increased transport time in the non-dominant vessel at week 1. When compared with ICG, the LI-COR dye produced much less severe effects on lymphatic function at the week 1 and 2 follow-up time points (Figure 8). The LI-COR dye produced a significantly lower percent change in transport time in both vessels at week 1 and in the non-dominant vessel in week 2 as compared to ICG. Similarly for packet frequency, the LI-COR dye produced a significantly reduced change at the week 1 time point in both vessels and at the 2 week time point in the non-dominant vessel as compared to ICG. Additionally, for packet velocity, the LI-COR dye produced a significantly reduced effect compared to ICG at the week 1 and 2 time points only in the non-dominant vessel. Finally, the LI-COR dye did not produce any significant changes in lymph node size at any time point.

## Discussion

### Vessel transport characteristics

The lymphatic vasculature is a complex network of interconnected vessels and nodes whose fluid transport capabilities are still not well-understood at the tissue level. This study represents a new effort toward quantifying the differential effects of multiple collecting lymphatic vessels draining a single tissue space, which will help to explain how the organ system functions as a whole in the context of fluid transport. We have provided a new framework for analyzing lymphatic function across multiple vessels to better assess cumulative lymphatic drainage of the interstitium. This framework will make NIR imaging more sensitive to lymphatic function, which may prove useful in distinguishing subtle changes between health and disease in the context of lymphatic research and the development of a point-of-care diagnostic.

Our results revealed that one vessel always produced fluorescence before the other with the difference in 10 cm transport time between the two vessels usually exceeding 20 s. In nearly every case, the first arrival vessel exhibited higher fluorescence intensity values, packet frequencies and packet velocities than the second arrival vessel, although both vessels consistently produced values in a range consistent with previously reported mesenteric lymph velocities (Dixon et al., 2006; Kassis, 2012) and packet frequencies and velocities in rat tail lymphatics (Weiler et al., 2012). We defined the first arrival vessel as dominant and the second arrival vessel as non-dominant in an effort to describe the observed difference in function between the two vessels. Importantly, the dominant vessel remained consistent across multiple injections in the same animal in the vast majority of cases, suggesting this phenomenon is repeatable and inherent to the function of that particular vessel network. Similar differential transport characteristics were also observed by Proulx et al. in two afferent collecting vessels of the mouse hind limb (Proulx et al., 2013), which indicates that our results may not be restricted to simply the rodent tail model and may be representative of general systemic lymphatic function. Future work will need to be done to address the relevance of these observations in rodents, to human lymphatic physiology.

There are several potential explanations for the observation of differential transport function between the two collecting vessels. Firstly, it is possible that preferential lymphatic drainage patterns exist such that for a given tissue space, fluid drainage is the primary responsibility of one single vessel, while any additional vessels in the area serve as overflow or reserve transport routes for large fluid loads. Preferential drainage patterns could be the result of regional variability in the location and distribution of different lymphatic vessel networks in the tissue space or the composition and organization of the interstitium at the injection site such that drainage into one collector over is favored over the other. Since our repeat injections in the same animal were performed as close to the original injection site as possible, either of these two potential explanations could account for the consistency of the dominant vessel across multiple injections. Although the utmost care was taken to ensure all injections were consistently given at the same angle, depth, and location, there is a substantial degree of inherent variability in the intradermal injection procedure, which could also be contributing to the observed differences in vessel function by differentially or inconsistently favoring drainage into one vessel over another. Furthermore, there were no detectable differences in vessel size within the spatial resolution limits of our imaging system, but fluorescence scattering makes accurate measurements of vessel diameter very difficult *in vivo*. Thus, it remains unclear whether subtle differences in vessel depth or vessel diameter may be a contributing factor favoring transport through one vessel over the other. Finally, the degree to which nodal resistance may contribute to the observed results is unknown and could be significant. Future work should specifically focus on the differences in function between multiple lymphatic vessels in several anatomic regions and under various experimental conditions to better characterize systemic lymphatic transport.

Our results also revealed a transient period during early fluorescence arrival in which lymphatic function was generally enhanced as compared to the subsequent steady-state period. This observation is not surprising when considering the bolus delivery and high albumin concentration of our injection. The duration of the transient arrival period was typically between 2–5 minutes, which is consistent with the small 10 μL volume of our injections and the time kinetics associated with responses to changes in Starling's forces observed in the microvasculature (Levick and Michel, 2010). After the initial response of the lymphatics to this change in the local interstitial fluid parameters that govern filtration, steady-state values of lymphatic transport were reached. Interestingly, more significant differences in function were observed between the dominant and non-dominant vessel during the transient arrival period than the steady-state period. This is consistent with the method we have utilized to categorize these two vessels (i.e., the vessel with a shorter arrival time is the dominant), as the arrival time is a metric that is reflective of vessel function during the initial transient response. Thus, the larger packet velocities and frequencies in the dominant vessel immediately after injection explain in part why transport time is shorter than in the non-dominant vessel.

These observations generally illustrate the importance of using a consistent framework to quantify lymphatic vessel function using NIR imaging. That is, we have shown that significant differences in transport characteristics are obtained by taking measurements during different periods following fluorophore injection or by quantifying different vessels draining the same tissue space. An optimal strategy for NIR quantification of vessel function remains undefined at this point, but future studies should be performed with careful consideration of the measurement framework, especially given that the selection of methodology for analysis can drastically affect results.

### Effects of nitric oxide

The effects of NO on vessel function further exemplify the importance of choosing an appropriate framework for transport quantification using NIR imaging. Our previous work showed that GTNO significantly reduced lymphatic drainage as evaluated by several function metrics (Weiler et al., 2012), but that analysis was limited to a single vessel and response time. Analyzing the effects of GTNO in the context of this more robust two-vessel, two-time-period framework may begin to provide a more broad understanding of the effects of NO on cumulative lymphatic drainage. Specifically, the results indicated that GTNO generally reduced function in both vessels, but more severely affected the non-dominant vessel.

The transport time metric, in particular, was very different between the two vessels after GTNO application. This metric incorporates extrinsic factors affecting uptake such as interstitial fluid pressure and matrix resistance as well as intrinsic factors affecting transport such as vessel contraction. Given that previous work has shown NO acts on lymphatics by strongly inhibiting vessel contractility (Gashev et al., 2002; Liao et al., 2011), these results could suggest a difference between the two vessels in the balance of intrinsic contractions vs. extrinsic factors in fluid transport following a large fluid load. That is, if preferential drainage patterns exist as we suspect, the increased interstitial fluid pressure after bolus injection would drive flow more favorably into the dominant vessel, while the non-dominant vessel would be forced to rely more exclusively on intrinsic contractions to transport fluid. The resultant vessel dilation following GTNO application would severely impair transport in the non-dominant vessel by inhibiting contractility. However, the effects would be much less severe in the dominant vessel because dilation would decrease vessel resistance, thus enhancing the effects of elevated interstitial fluid pressure driving flow into the vessel. This could help to explain why NO more significantly affected the non-dominant vessel, especially in the transport time metric.

Alternatively, these observations could be the result of secondary effects of NO on lymph formation, which could differentially affect uptake and subsequent transport in the two vessels. The lymphatic community has recently become very interested in the modulating effects of NO on lymphatic function in health and disease (Bohlen et al., 2011; Liao et al., 2011; Scallan and Davis, 2013), and future work devoted to a more thorough characterization of NO on lymphatic network drainage would be well-warranted.

### Effects of NIR fluorophores

In this study, we have shown for the first time with NIR imaging that injection of ICG contributed to long-term decreases in lymphatic function. The results of the long-term follow-up screenings revealed that ICG was retained in the local tissue space for approximately 2 weeks after injection, and lymphatic transport was reduced during this period. There was a fairly dramatic decrease in function at the 1 week time point in which all metrics were reduced in both vessels, but week 2 produced a less severe reduction in function and was limited exclusively to the non-dominant vessel. Lymph nodes were also enlarged at 1 and 2 weeks after injection, but interestingly, there was not a significant difference in size between lymph nodes of the dominant and non-dominant vessel. Very importantly, control animals in which the initial injection contained albumin but lacked ICG did not produce any significant change in function or lymph node size at any time point. Since the control injections were the same volume and given in the same manner as the ICG injections, this data suggests that the observed decreases in lymphatic function following initial injection of ICG were due to the fluorophore itself rather than the injection procedure or any other constituent of the injection solution.

Fortunately, the effects of ICG appear to be reversible since all of the functional metrics returned to baseline levels in both vessels and lymph nodes returned to normal size at the week 4 time point. Despite the data showing a functional decrease in lymphatic transport after ICG injection, it is difficult to determine whether the observed effect sizes are substantial enough to warrant physiological concern. Although lymph nodes showed signs of enlargement after ICG injection, the level of hypertrophy was not nearly as large as the 20- to 30-fold increase in nodal weight seen in models of chronic inflammation (Ezaki et al., 2001; Baluk, 2005). No detectable edema was observed in the tail of any of the animals at any point during the time course, which suggests that ICG does not decrease lymphatic drainage to the point of causing significant fluid stagnation or inflammation. It does not appear that ICG needs to be treated as a health concern at least at frequencies that don't exceed once per month, but care should be taken in interpreting NIR functional data with this fluorophore, especially in the context of repeat injections. Repeat injections with ICG may need to be spaced as far apart as 4 weeks in order to avoid erroneous measurements in function, although a similar investigation of time-course effects of ICG in humans would be valuable for the continued development of a point-of-care diagnostic.

The observed decreases in lymphatic function are consistent with the investigation of ICG in isolated lymphatic vessels by Gashev et al. (2010). Our result, that reduced function appeared to coincide with ICG retention, is similar to their observation that vessel contraction was inhibited even beyond the period of ICG washout. Gashev et al. was able to show ICG binds to the endothelial cell layer in the isolated vessel setup, but in our study, ICG was most visibly retained at the injection site. A small amount of fluorescence was visible in the collecting vessels for 1–2 days immediately after the injection, but the intensity quickly dropped below the limit of detection due to scattering effects and a low concentration of dye. With our imaging system the limit of detection of ICG in tail collecting vessels is in the micromolar range, which is consistent with concentrations used by Gashev et al. It is difficult, therefore, to make strong conclusions from our data regarding the mechanism by which ICG affected lymphatic function. At the very least, however, the injection site would have been constantly releasing small amounts of ICG into the lymphatic vessels over the course of the retention period, which could have contributed to the long duration of decreased function. Given the evidence of ICG toxicity with prolonged exposure in the retina (Ikagawa et al., 2005), it is possible that the decrease in lymphatic function could also be a result of ICG reported mild toxicity.

One limitation of our study is that we only used one concentration of ICG for injection. Although the volume of the injection is very small, the concentration is optimized for fluorescence visualization rather than consideration of vessel function. It is possible that a more dilute solution, while negatively influencing fluorescence intensity, may contribute to shorter retention periods and have less of a long-term impact on function.

An additional investigation by Aldrich et al. did not detect a change in lymphatic function in response to ICG injections (Aldrich et al., 2012), but these results do not necessarily conflict with ours and there are several explanations for these results. Firstly, it should also be noted that our initial baseline readings for ICG and the LI-COR IRDye 800CW are similar to those reported by the authors. The principal difference between the two studies is the time-point of interest after injection. Aldrich et al. examined lymphatic function effects immediately after injection while we used this value as a baseline and instead focused on time course differences 1, 2, and 4 weeks later. Given that ICG has been shown accumulate in the intracellular space for a period beyond 24 h (Fickweiler et al., 1997; Abels et al., 2000), time may be a necessary component for the cumulative dose to affect vessel function. Another difference between our study and that of Aldrich et al. is the above-mentioned framework used for analysis of NIR imaging. This framework was critical to generating our results, especially since more profound effects were seen in the non-dominant vessel and during the arrival period initially after injection. Finally, our study was performed in the rat tail, while Aldrich et al. used the mouse inguinal-to-axillary model, and it remains unclear if and how lymphatic function may vary between the two regions and the two species.

The functional results of the LI-COR IRDye 800CW PEG provide an interesting comparison to ICG. The dye produced a mild decrease in lymphatic function at week 1, but it did not contribute to a deterioration of function nearly as severe as ICG and did not affect lymph node size. These results are not meant to indicate that the LI-COR IRDye is an optimal solution for NIR lymphatic imaging, although it appears to have less of a long-term effect on lymphatics than ICG, but rather to illustrate that the choice of fluorophore impacts NIR lymphatic functional measurements. Thus, optimizing the tracer of choice to minimize its biological impact is warranted, particularly in the context of longitudinal lymphatic imaging. The difference in functional response between ICG and the IRDye may suggest that the chemical composition and three-dimensional structure of the fluorophore either directly modulates lymphatic transport or indirectly affects lymphatic function through eliciting an immune response (Liao et al., 2011), especially given the observed differential effects on lymph node size between the two dyes in this study. The NIR lymphatic imaging community has been directing research toward the development and optimization of fluorophores specifically for the purpose of lymphatic imaging (Proulx et al., 2010, 2013; Davies-Venn et al., 2011). While these efforts have concentrated primarily on using dyes with higher quantum yields than ICG or altering the size of the carrier molecule to optimize lymphatic uptake, future work in the development of new probes should also focus on the long-term effects of the dyes on local lymphatic and immune function. Repeat injections, either in the context of research or a point-of-care diagnostic will need to have no effect on lymphatic function in order to obtain meaningful and consistent results.

### Conflict of interest statement

The authors declare that the research was conducted in the absence of any commercial or financial relationships that could be construed as a potential conflict of interest.
